# Culture-Negative Lemierre’s Syndrome: A Case Report of Septic Thrombophlebitis With Multi-organ Involvement

**DOI:** 10.7759/cureus.88341

**Published:** 2025-07-20

**Authors:** Mohannad Ali, Saud Abdullah, Abeer AlHaj

**Affiliations:** 1 Internal Medicine, DubaiHealth, Dubai, ARE

**Keywords:** clinical diagnosis, culture negative, lemierre's, multiorgan dysfunction, septic thrombophlebitis

## Abstract

Lemierre’s syndrome is a rare and potentially life-threatening condition characterized by septic thrombophlebitis of the internal jugular vein, typically following an oropharyngeal infection. We report a case of a young, previously healthy patient who presented with sepsis and multi-organ involvement, later diagnosed with right internal jugular vein thrombosis. Despite negative blood cultures, clinical features supported a diagnosis of Lemierre’s syndrome. The patient was treated with broad-spectrum antibiotics and anticoagulation, later complicated by hemoptysis requiring adjustment of therapy. This case brings attention to the diagnostic challenges in culture-negative Lemierre’s syndrome and underscores the importance of early recognition, imaging, and individualized management, particularly in the absence of standardized guidelines for antibiotic and anticoagulant duration.

## Introduction

Lemierre’s syndrome is a rare but potentially life-threatening condition typically arising from oropharyngeal infections that lead to bacteremia, septic thrombophlebitis of the internal jugular vein (IJV), and septic emboli. Originally described by Lemierre in 1936, the condition predominantly affects adolescents and young adults, with an estimated incidence of 3.6 to 5 cases per million annually, rising to 14.4 per million in individuals aged 15-24 years [1-3]. Although *Fusobacterium necrophorum* remains the most common etiological agent, culture-negative cases and atypical pathogens are increasingly reported [4]. Typical management includes prolonged antibiotic therapy and consideration of anticoagulation, yet no formal guidelines exist to define optimal treatment duration. Due to its rarity, clinicians may be unfamiliar with the varied presentations of Lemierre’s syndrome, particularly when blood cultures fail to isolate the typical pathogens. Such culture-negative cases can present significant diagnostic challenges and contribute to delays in initiating appropriate treatment. Here, we report a case of a 30-year-old man who presented with sepsis, multi-organ dysfunction, and septic pulmonary emboli, later diagnosed with IJV thrombosis, in the absence of a clearly identified causative pathogen. This case underscores the need for a high index of suspicion for Lemierre’s syndrome, even in the absence of classical microbiological or radiological findings.

## Case presentation

A 30-year-old male, previously healthy with no known comorbidities, presented to the emergency department with a one-week history of fever, generalized myalgia, fatigue, and odynophagia. Associated with a four-day history of anorexia, headache, mild generalized abdominal pain, and three episodes of vomiting containing food content, one of which was associated with streaks of blood. The patient had not received any antibiotics prior to presentation in the last three months, with no significant history of lifetime antibiotics use mentioned by the patient, and had only taken symptomatic treatments for the current presentation. He was a current smoker with a 10-year history of tobacco use.

On initial assessment in the emergency department, the patient appeared acutely ill, requiring 3 liters of supplemental oxygen through a nasal cannula to maintain oxygen saturation above 94%. He was tachypneic with a respiratory rate of 28 breaths per minute and tachycardic with a heart rate ranging from 110 to 120 beats per minute. He exhibited scleral icterus. Oropharyngeal examination revealed erythema without tonsillar enlargement, exudates, or uvular deviation. Chest auscultation revealed bilateral infrascapular crackles posteriorly, with no detectable cardiac murmurs. Abdominal examination was notable for diffuse mild tenderness without guarding, rigidity, or organomegaly. Peripheral pulses were intact. The patient had localized tenderness on the right side of the neck over the anterior triangle of the neck, without overlying skin changes or palpable lymphadenopathy.

Initial laboratory investigations revealed a hemoglobin level of 12.3 g/dL, leukocytosis with a white blood cell count of 15,500/µL, and severe thrombocytopenia with a platelet count of 9,000/µL. Inflammatory markers were markedly elevated, with a C-reactive protein (CRP) of 186.5 mg/L and procalcitonin (PCT) of 59.28 ng/mL. Renal function tests showed significant impairment, with a serum creatinine of 6.62 mg/dL, urea of 221 mg/dL, and an estimated glomerular filtration rate (eGFR) of 10.7 mL/min/1.73 m². Liver function tests demonstrated total bilirubin of 5.46 mg/dL (direct bilirubin 4.51 mg/dL, indirect bilirubin 0.95 mg/dL), with Alanine Aminotransferase (ALT) at 25 U/L, aspartate aminotransferase (AST) at 28 U/L, alkaline phosphatase (ALP) at 154 U/L, and gamma-glutamyl transferase (GGT) at 44 U/L. Electrolyte panel showed sodium of 134 mmol/L, potassium 4.1 mmol/L, and bicarbonate 18.0 mmol/L. Pro-B-type natriuretic peptide (Pro-BNP) was elevated at 2,224 pg/mL.

Other negative or unremarkable results included coagulation profile, urine analysis, urine culture, direct Coombs’s test, lipase, lactate dehydrogenase, creatine phosphokinase (CPK), troponin, CK-MB, HIV antigen/antibody, and respiratory viral panel (influenza A, influenza B, RSV, and SARS-CoV-2 PCR). Autoimmune and vasculitis screening, including antinuclear-antibodies (ANA), complement levels (C3, C4), anti-dsDNA, extractable nuclear antigen (ENA) profile, proteinase-3 (PR3) antibody, and myeloperoxidase (MPO) antibody, were all negative. Peripheral blood smear demonstrated reactive thrombocytopenia and normochromic, normocytic anemia, with no additional abnormalities noted. Testing for dengue fever was negative. Three sets of blood cultures collected at presentation showed no growth.

**Figure 1:**
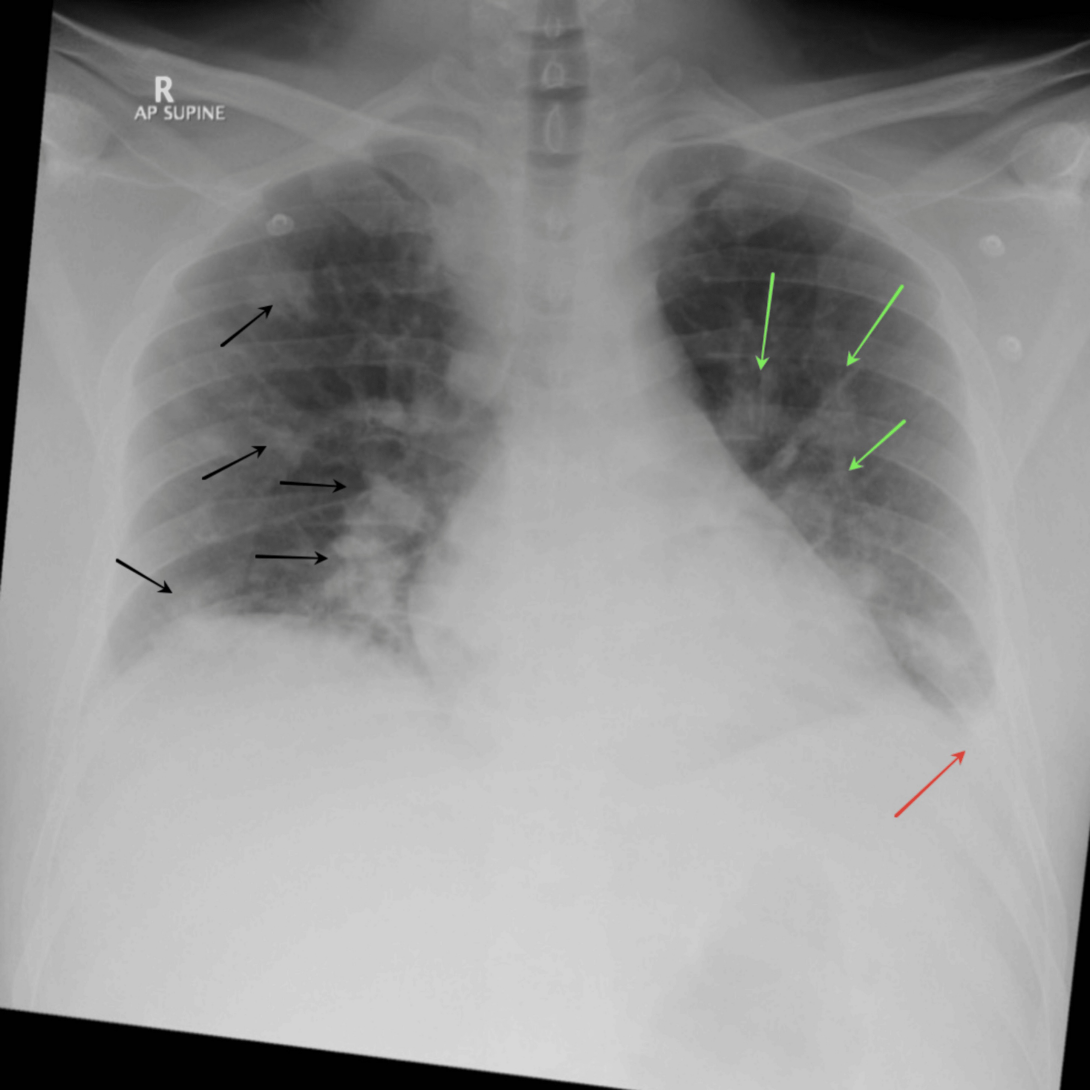
X-ray chest upon first presentation to the emergency department (supine anteroposterior view expiratory phase) X-ray of the chest revealed evidence of multifocal pneumonitis in bilateral lower zones and right upper and mid zones (black arrows) along with a few infiltrates in the left mid zone (green arrows) and blunting of the left costophrenic angle (red arrow).

**Figure 2:**
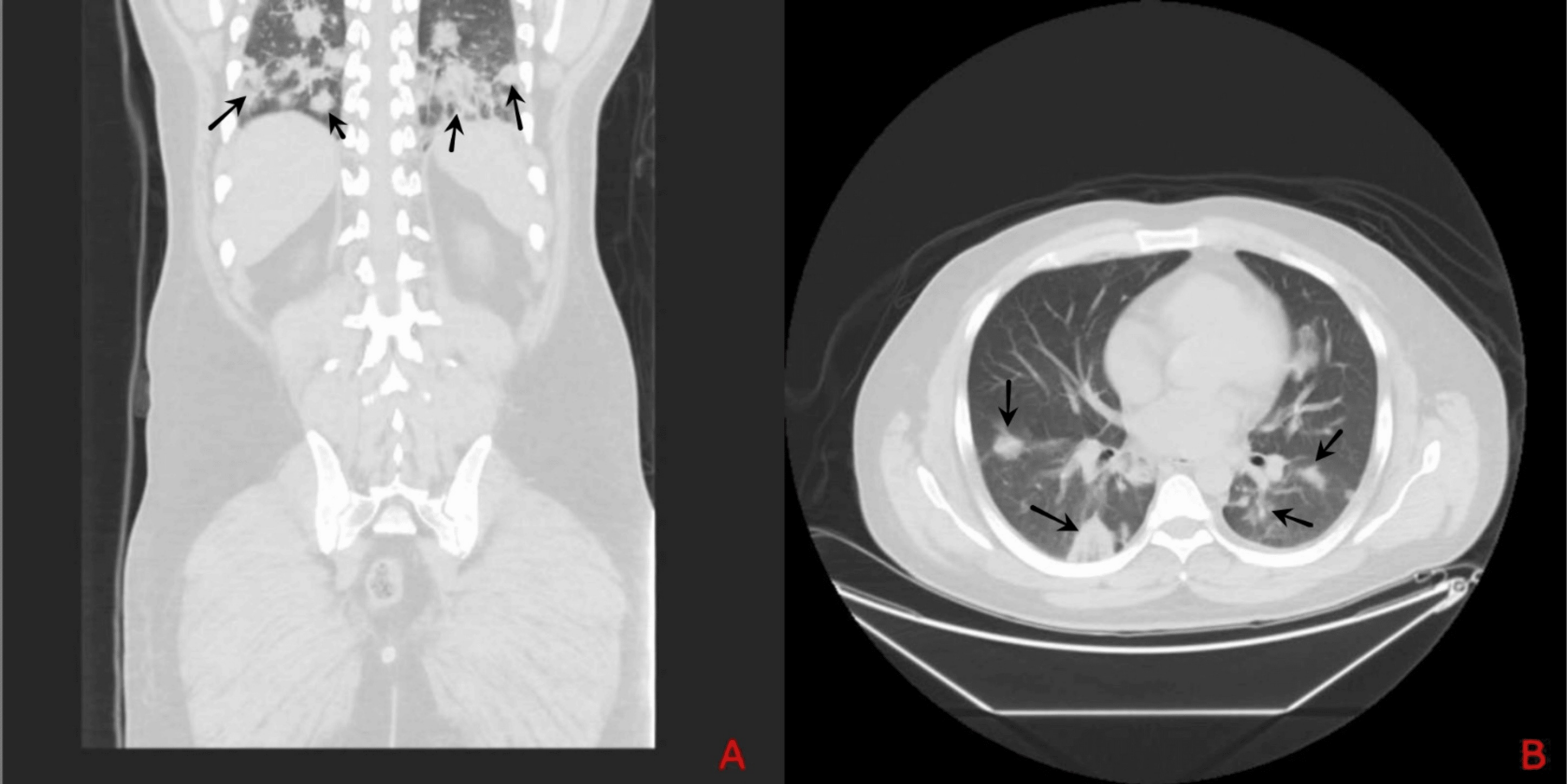
Axial & coronal sections of computed tomography (CT) scan of the abdomen showing multiple patchy areas of consolidation in bilateral lung bases with coalescence suggestive of multifocal pneumonitis with few round opacities (black arrows)

Bedside transthoracic echocardiography (TTE) was unremarkable, showing no evidence of valvular abnormalities or intracardiac thrombi. Chest radiograph showed multifocal pneumonitis with infiltrates noted in the left mid and lower lung zones and the right upper and mid zones (Figure 1). A non-contrast computed tomography (CT) of the abdomen revealed bilateral renal enlargement with increased parenchymal thickness and mild perirenal fat stranding. In addition, the visualized lower segments of the lung showed multiple patchy areas of consolidation in bilateral lung bases and a few round opacities suggestive of possible septic emboli (Figures 2A-2B). 

The patient was admitted under internal medicine with a preliminary diagnosis of sepsis of unknown origin complicated by multi-organ dysfunction. Empiric broad-spectrum antibiotics were initiated, including intravenous piperacillin-tazobactam and renally adjusted intravenous levofloxacin, along with supportive care and intravenous fluid resuscitation. Piperacillin-tazobactam and levofloxacin were chosen to provide broad-spectrum coverage, including gram-negative bacilli, anaerobes, Pseudomonas aeruginosa, and atypical organisms, given the patient’s presentation with severe sepsis and an unclear infectious source. Levofloxacin was specifically included to enhance coverage for atypical pathogens not reliably targeted by piperacillin-tazobactam. A single dose of vancomycin (1 g) was also administered early to provide methicillin-resistant *Staphylococcus aureus* (MRSA) coverage; however, it was discontinued as the patient was deemed low risk for MRSA and there were no clinical or microbiological indicators suggestive of MRSA infection. The antimicrobial regimen was reassessed as the clinical picture evolved, and a decision was made to continue piperacillin-tazobactam and levofloxacin due to marked clinical and biochemical improvement. De-escalation was not pursued, as blood cultures remained negative.

**Figure 3:**
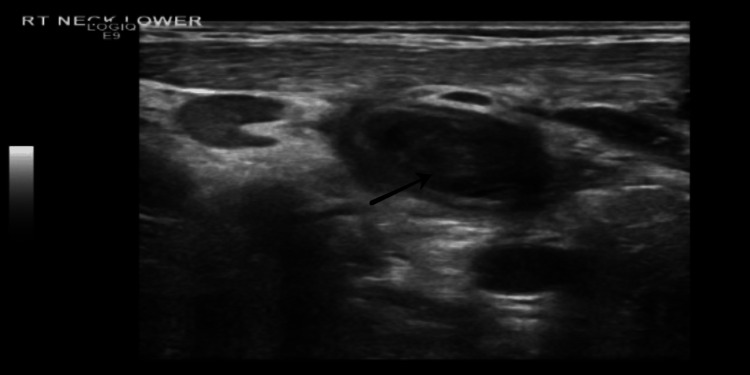
Ultrasound of the neck showing a thrombus in the internal jugular vein (black arrow)

The patient demonstrated marked clinical and biochemical improvement; however, he continued to complain of right-sided neck pain. A careful re-examination of the neck revealed a tender cord-like structure over the tender area. Doppler ultrasonography of the neck confirmed thrombosis of the right IJV as shown in Figure 3. And thus the patient was diagnosed with Lemierre's syndrome six days after admission. 

Following a thorough discussion regarding the risks and benefits of anticoagulation in the absence of definitive guidelines, the patient opted to begin oral anticoagulation and was discharged on rivaroxaban 15 mg twice daily. He received 11 days of intravenous piperacillin-tazobactam and intravenous levofloxacin as an inpatient and was discharged on oral levofloxacin for 3 additional days. The duration of antibiotics was decided based on expert opinion from the internal medicine consultant physician, along with clinical and biochemical improvement as shown in Table 1.

**Table 1:** Comparison of Laboratory Test Values on Admission and at Discharge (Day 11)

	Reference Range	On Admission	On discharge
White blood cells	3.6 - 11.0 × 10^3^/µL	15,500/µL	6,400/ µL
Hemoglobin	13.0 - 17.0 g/dL	12.3 g/dL	10.9 g/dL
Platelets	150 - 410 × 10^3^/µL	9,000/µL	259,000/ µL
C-reactive protein	< 5.0 mg/L	186.5 mg/L	4.0 mg/L
Procalcitonin	<0.05 ng/mL	59.28 ng/mL	0.06 ng/mL
Creatinine	0.7 - 1.2 mg/dL	6.62 mg/dL	1.10 mg/dL
Glomerular filtration rate	>60 mL/min/1.73 m²	10.7 mL/min/1.73 m²	92.60 mL/min/1.73m²
Urea	12 - 40 mg/dL	221 mg/dL	25 mg/dL
Bilirubin, Total	0 - 1.0 mg/dL	5.46 mg/dL	1.04 mg/dL
Alkaline phosphatase	40 - 129 U/L	154 U/L	138 U/L

Five days following discharge, the patient returned to the emergency department with an episode of frank hemoptysis and a hemoglobin drop of 1 g/dL (from 10.9 g/dL to 9.9 g/dL). He was readmitted with a presumed diagnosis of anticoagulation-associated hemoptysis. Anticoagulation was temporarily held for the first 48 hours. After a thorough reassessment and shared decision-making along with the vascular surgery and pulmonology teams, the patient agreed to resume anticoagulation with a therapeutic dose of low-molecular-weight heparin (LMWH) for 1 week - the first 3 days as an inpatient and the remaining 4 days post-discharge. This was followed by re-initiation of rivaroxaban 20 mg once daily for a duration of 3 months. He remained stable without further hemoptysis or any further hemoglobin decline and was discharged after a couple of days of observation. At the time of discharge, his hemoglobin was 9.7 g/dL, and he was clinically well with no recurrence of neck pain or bleeding. The patient, however, did not follow up for repeat imaging of the IJV or any further tests.

## Discussion

The clinical features observed in our patient are consistent with Lemierre syndrome despite negative blood cultures. Although the classic definition of Lemierre’s syndrome involves IJV thrombosis secondary to oropharyngeal infection with Fusobacterium necrophorum, the term is commonly applied in clinical practice to culture-negative cases with similar pathophysiology and presentation. Fusobacterium necrophorum remains the classic causative pathogen; however, culture-negative cases are reported in up to 12-38% of patients, often due to prior antibiotic exposure [2,4]. Other pathogens have also been identified, either as sole isolates or mixed with Fusobacterium necrophorum. These include various *Bacteroides* species, *Peptostreptococcus*, Group B and C *Streptococcus*, *Streptococcus oralis*, *Staphylococcus epidermidis*, *S. aureus*, *Enterococcus* species, *Proteus mirabilis*, *Eubacterium*, *Eikenella corrodens*, *Lactobacilli*, and *Candida* species [4].

The most reported presenting complaints include fever, sore throat, neck pain or swelling, pleuritic chest pain, and dyspnea [4]. Atypical presentations have also been documented, encompassing hemoptysis, cranial nerve palsies, and abdominal symptoms, emphasizing the syndrome's variety in clinical presentation [4,5]. This diversity can contribute to diagnostic delays.

Antibiotic therapy remains the cornerstone of management. Empiric regimens should provide broad coverage targeting anaerobic and typical oropharyngeal flora. Recommended agents include beta-lactam/beta-lactamase inhibitor combinations such as piperacillin-tazobactam, ceftriaxone plus metronidazole, or carbapenems [6,7]. In patients presenting with hemodynamic instability, features of severe disease, or established risk factors for MRSA, empiric antibiotic therapy should include agents effective against MRSA. Although there is no consensus on treatment duration, courses commonly range from 2 to 6 weeks and are tailored based on clinical response and the extent of embolic disease.

The role of anticoagulation in Lemierre syndrome remains controversial. Observational studies and case series suggest potential benefits in cases with thrombus propagation or persistent emboli; however, many patients recover without anticoagulation, emphasizing the need for individualized risk-benefit assessment. A population-based study by Nygren et al. found no significant difference in clinical outcomes such as thrombosis progression, septic emboli, or 30-day mortality between patients treated with therapeutic anticoagulation and those who received prophylactic or no anticoagulation. Importantly, anticoagulation was well tolerated, with a low incidence of bleeding events, suggesting it may be safe in selected cases [8-10]. Prospective research is necessary to define clear guidelines regarding the indications, timing, and duration of anticoagulation in this condition. Our patient was started on oral anticoagulation after careful multidisciplinary discussion but required temporary cessation and adjustment following an episode of anticoagulation-associated hemoptysis, underscoring the challenges of managing thrombotic and bleeding risks in this context.

Xie et al. highlight the heterogeneity of clinical presentations and disease severity, with antibiotic choices typically reflecting infection severity and local practice patterns. Their review further emphasizes individualized anticoagulation decisions, often guided by thrombus extent and embolic complications [5]. Compared to their cases, our patient exhibited multi-organ involvement with renal impairment and pulmonary septic emboli, features consistent with severe disease manifestations described in the literature.

Given the syndrome’s rarity and variety in clinical presentation, increased clinical awareness, early broad-spectrum antibiotic initiation, and timely imaging are essential for favorable outcomes. Our case underscores the importance of thorough physical examination and repeat imaging, which confirmed IJV thrombosis and guided subsequent management.

## Conclusions

Lemierre’s syndrome is often referred to as a “forgotten disease”, given its rarity in modern clinical practice and the nonspecific nature of its initial presentation. However, it remains a serious and potentially fatal condition. Clinicians should maintain a high degree of suspicion in patients presenting with sepsis, neck pain, and evidence of thromboembolic phenomena, especially in the absence of an identified source. Early diagnosis, appropriate antimicrobial therapy, and a thoughtful approach to anticoagulation are essential for optimizing outcomes.
